# Cyclophosphamide- metabolizing enzyme polymorphisms and survival outcomes after adjuvant chemotherapy for node-positive breast cancer: a retrospective cohort study

**DOI:** 10.1186/bcr2570

**Published:** 2010-05-10

**Authors:** Priya P Gor, H Irene Su, Robert J Gray, Phyllis A Gimotty, Michelle Horn, Richard Aplenc, William P Vaughan, Martin S Tallman, Timothy R Rebbeck, Angela DeMichele

**Affiliations:** 1 Current address: Center for Cancer and Hematologic Disease, Executive Mews Office Complex, 1930 E. Route 70, Suite V-107, Cherry Hill, NJ 08003, USA; 2 Department of Obstetrics and Gynecology, University of California, San Diego, Moores UCSD Cancer Center, 3855 Health Sciences Drive, Dept. 0901, La Jolla, CA 92093-0901, USA; 3 Department of Epidemiology and Biostatistics, University of Pennsylvania, Philadelphia, PA 19104, USA; 4 Abramson Cancer Center of the University of Pennsylvania, 3400 Civic Center Blvd, Philadelphia, PA 19104, USA; 5 Eastern Cooperative Oncology Group, Frontier Science, 900 Commonwealth Avenue, Boston, MA 02215, USA; 6 Division of Oncology, Children's Hospital of Philadelphia, Room 916G, ARC 3615 Civic Center Blvd, Philadelphia, PA 19104, USA; 7 Division of Hematology-Oncology, Northwestern University Feinberg School of Medicine, 676 N St Clair St, Ste 850, Chicago, IL 60611, USA; 8 Robert H. Lurie Comprehensive Cancer Center, Clinical Cancer Center, Galter Pavilion 675 North St Clair, 21st Floor, Chicago, Illinois 60611, USA; 9 Department of Hematology & Oncology, University of Alabama at Birmingham, 1900 University Blvd, Ste THT 541, Birmingham, AL 35294, USA; 10 Rena Rowan Breast Cancer Center, Division of Hematology-Oncology, Department of Medicine,, University of Pennsylvania Health System, Perelman Center, 3400 Civic Center Boulevard, Philadelphia, PA 19104, USA

## Abstract

**Introduction:**

Cyclophosphamide-based adjuvant chemotherapy is a mainstay of treatment for women with node-positive breast cancer, but is not universally effective in preventing recurrence. Pharmacogenetic variability in drug metabolism is one possible mechanism of treatment failure. We hypothesize that functional single nucleotide polymorphisms (SNPs) in drug metabolizing enzymes (DMEs) that activate (CYPs) or metabolize (GSTs) cyclophosphamide account for some of the observed variability in disease outcomes.

**Methods:**

We performed a retrospective cohort study of 350 women enrolled in a multicenter, randomized, adjuvant breast cancer chemotherapy trial (ECOG-2190/INT-0121). Subjects in this trial received standard-dose cyclophosphamide, doxorubicin and fluorouracil (CAF), followed by either observation or high-dose cyclophosphamide and thiotepa with stem cell rescue. We used bone marrow stem cell-derived genomic DNA from archival specimens to genotype CYP2B6, CYP2C9, CYP2D6, CYP3A4, CYP3A5, GSTM1, GSTT1, and GSTP1. Cox regression models were computed to determine associations between genotypes (individually or in combination) and disease-free survival (DFS) or overall survival (OS), adjusting for confounding clinical variables.

**Results:**

In the full multivariable analysis, women with at least one CYP3A4 *1B variant allele had significantly worse DFS than those who were wild-type *1A/*1A (multivariate hazard ratio 2.79; 95% CI 1.52, 5.14). CYP2D6 genotype did not impact this association among patients with estrogen receptor (ER) -positive tumors scheduled to receive tamoxifen.

**Conclusions:**

These data support the hypothesis that genetic variability in cyclophosphamide metabolism independently impacts outcome from adjuvant chemotherapy for breast cancer.

## Introduction

Women with node-positive breast cancer typically receive cyclophosphamide-based adjuvant chemotherapy, but a significant proportion of these women relapse and ultimately die of their disease. A growing body of literature suggests that individual variability in drug metabolism impacts pharmacodynamics and subsequent efficacy [1]. Functional single nucleotide polymorphisms (SNPs) in drug metabolizing enzymes (DMEs) are a major determinant of this variability [2].

**Figure 1:**
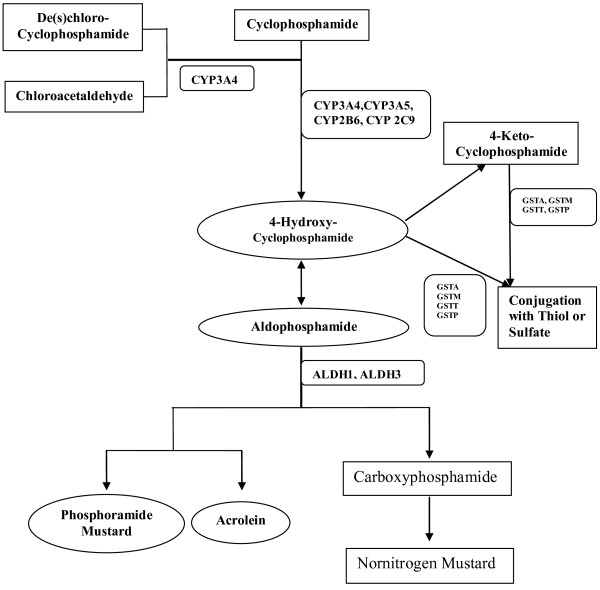
**Cyclophosphamide metabolism**.

Cyclophosphamide is administered as an inactive prodrug that must undergo activation through phase I metabolism by cytochrome P450 (CYP) enzymes 2B6, 3A4, 3A5, and 2C9 and phase II inactivation primarily through conjugation with a thiol or sulfate via glutathione S-transferases (GSTs) alpha, mu, theta or pi as shown in Figure 1. The active metabolite, 4-hydroxy-cyclophosphamide diffuses into cancer cells [3] and is responsible for cyclophosphamide's alkylating ability [4,5]. Functional SNPs in these enzymes impact enzyme activity and metabolite levels. Several small prior studies, including our own, support the hypothesis that functional SNPs in these phase I and phase II enzymes impact clinical outcome in breast cancer [6-8].

To further evaluate this hypothesis, we examined whether cyclophosphamide-DME SNPs were independently associated with disease-free or overall survival (DFS, OS) in a cohort of women enrolled in a multicenter, randomized, adjuvant breast cancer chemotherapy trial.

## Materials and methods

We performed a retrospective cohort study utilizing genomic DNA derived from hematologic circulating or bone-marrow-derived stem cells and clinical data from breast cancer patients enrolled on Intergroup Trial 0121 (E2190/SWOG9061/CALGB 9496), a multicenter trial of high dose vs. standard dose adjuvant chemotherapy. Patients were included in the current study if they were enrolled in INT-0121, informed consent was confirmed, and archival peripheral blood or bone marrow stem cells were available for genomic DNA extraction and subsequent genotyping. This study is in compliance with the Helsinki Declaration and was performed with the approval of the University of Pennsylvania Institutional Review Board and the Eastern Cooperative Oncology Group (ECOG) Executive Committee.

Results of E2190/INT-0121 trial have been published previously [9]. Briefly, 540 patients with ≥10 positive lymph nodes received conventional adjuvant therapy with four cycles of cyclophosphamide (C; 100 mg/m2, orally, Days 1 to 14), doxorubicin (A; 30 mg/m2, intravenously, Days 1, 8), and fluorouracil (F; 500 mg/m2, intravenously, Days 1, 8) followed by randomization to either observation or receipt of high-dose chemotherapy (HDC: cyclophosphamide (6 gm/m2) and thiotepa (800 mg/m2) over a four-day period) with hematopoietic stem cell reinfusion. Adjuvant tamoxifen was recommended for patients with estrogen receptor (ER) positive tumors, although receipt of this medication was not tracked in the study database.

The protocol specified hematopoietic stem cell collection at the completion of standard CAF for all patients on the study. Specimens not utilized for autologous reinfusion were stored at -80 C at the ECOG Pathology Core Facility (Chicago, IL, USA). The original INT-0121 consent form included language specifying that residual biological specimens would be used for future breast cancer research. The ECOG Statistical Center (Boston, MA, USA) performed additional follow-up and de-linked patient identifiers from the clinical data used in this analysis. The primary endpoint for this study was DFS, defined as time from randomization to earliest recurrence, new breast cancer, or death. The secondary endpoint was overall survival, defined as time from randomization to death [9]. All survival times were censored at time of last contact or on 1 August 2005 if subjects were alive and disease-free at that time.

We selected 15 SNPs (CYP2B6*4, CYP2B6*5, CYP2B6*6, CYP2B6*7, CYP2B6*9, CYP2C9*2, CYP2C9*3, CYP3A4*1B, CYP3A5*3, CYP3A5*6, CYP2D6*4, GSTM1, GSTT1, GSTP1*B, GSTP1*C) in eight genes for inclusion by first identifying cyclophosphamide-metabolizing enzyme polymorphisms that were associated with functional effects on enzyme expression, levels or activity, then excluding those in which the expected prevalence of the combination of alleles for a particular gene was ≤ 10% of the general population, since these constituted a minute fraction of cyclophosphamide metabolism and significant effects for these SNPs were unlikely to be detectable with the sample size available. All variants were hypothesized to result in decreased enzyme function [10]. Of note, genotyping for CYP2D6*4 was included to account for any possible differential effect of variable tamoxifen metabolism on outcome among patients whose tumors were estrogen-receptor positive.

The ECOG Pathology Core Facility at Northwestern University extracted DNA from hematologic stem cells with the EZ1 system (Qiagen, Inc, Hilden, NW, Germany). Genotyping was performed at the University of Pennsylvania. GSTM1 and GSTT1 homozygous null mutations were detected using a method previously described, using a 4% metaphor agarose gel [11,12]. The remainder of genotyping was determined by PyroSequencing (CYP2C9*3, CYP3A4*1B, CYP3A5*3, CYP3A5*6, GSTP1*B, GSTP1*C) (Biotage, Charlottesville, VA, USA, Applied Biosystems, Foster City, CA, USA)) and Taqman Real-Time PCR assays on the MJ Research Chromo4 (Bio-Rad) platform (CYP2B6*4, CYP2B6*5, CYP2B6*6, CYP2B6*7, CYP2B6*9, CYP2C9*2, CYP2D6*4). The technician performing genotype assays was blinded to all clinical and outcome data.

Polymorphisms were examined individually and in genotype groups defined based on our prior work [6]. With regard to CYP2B6, assignment of genotype was carried out as follows: carriers of the A785G mutation alone were designated CYP2B6*4; carriers of the C1459T mutation alone were designated CYP2B6*5; carriers of the combination of A785G and G516T were designated CYP2B6*6; carriers of the combination of A785G, G516T, and C1459T were designated CYP2B6*7; and carriers of the G516T mutation alone were designated CYP2B6*9 (data not shown). Because of the similarly anticipated directions of effect and the lack of power to perform comparisons with each polymorphism, dichotomous CYP2B6, CYP2C9, and GSTP1 variables were created based on genotype, where groupings consisted of all wild-type versus carriers of any variant.

To validate our pilot work assessing combined effects of variants in both activating (CYP) and metabolizing (GST) enzymes, we also classified subjects into three groups (favorable, intermediate and unfavorable) based on their CYP3A4*1B, CYP3A5*3, GSTM1 and GSTT1 genotypes [6]. The favorable group was comprised of subjects with no variant in either CYP3A4 or CYP3A5 and null at both GSTM1 and GSTT1. The unfavorable group was comprised of subjects who were variant in either CYP3A4 or CYP3A5 and non-null at both GSTM1 and GSTT1. The intermediate group comprised all other CYP/GST combinations. The groups were hypothesized to have varying serum concentrations of active cyclophosphamide metabolites based on known functional significance of the genetic variants.

STATA (Release 9, Stata Corporation, College Station, TX, USA) and R (Version 2.3.1, R Foundation for Statistical Computing, Vienna, Austria) software were used for statistical analysis. Pearson's chi-squared or exact tests for small samples were used to compare proportions. Survival curves were generated using the Kaplan-Meier method [13] and were compared using the log-rank test. Cox regression models were computed to determine the hazard ratio associated with each genotype, genotype group or clinical variable and both DFS and OS. Indicator variables for categorical variables included a category for missing data. The Grambsch-Therneau test was used to test the proportional hazards assumption [14]. Multivariable Cox models for DFS and OS were developed by including all genotype and clinical variables. All tests of significance were two-sided, with alpha = 0.05.

## Results

A total of 433 peripheral blood or bone marrow stem cell specimens for genomic DNA from 540 patients originally enrolled in INT-0121 were identified for study from the ECOG Pathology Core Facility. Fifty-two samples could not be linked to clinical data and follow-up; 31 samples were duplicates. Thus, a final study cohort of 350 subjects (65% of parent trial) was available for this analysis.

Clinical characteristics of all 540 INT-0121 patients as well as those genotyped (n = 350) appear in Table 1. Genotyped subjects were more likely to be enrolled in the CAF + HDC arm compared to the non-genotyped group (57% v. 38%, *P *< 0.001), likely due to the fact that some patients randomized to the observation arm did not have bone marrow or stem cells collected post-adjuvant therapy, resulting in more bone marrow/stem cell samples collected for those patients randomized to the transplant arm than to the observation arm. Overall, women in the genotyped cohort had significantly shorter DFS than women in the study overall and those not genotyped, but did not differ with respect to OS.

**Table 1:** Study population and comparison to full trial

Characteristic	Genotyped Study Cohort N = 350	Full E2190/INT-0121 Cohort N = 540	*P*-value^1^
	
	Median (IQR)^2^	Median (IQR)^2^	
Age	45 (39 to 50)	44 (38 to 50)	0.35
Axillary LN positive	14 (11 to 19)	14 (11 to 18)	0.13
Tumor size, cm	3.5 (2.1 to 5.0)	3.5 (2.1 to 5.0)	0.90
Median follow-up years	9.8 (8.3 to 11.2)	9.7 (8.1 to 11.4)	0.57
	Percent (95% CI)	Percent (95% CI)	
Postmenopausal	31 (26 to 35)	29 (25 to 33)	0.22
Race - Caucasian	90 (87 to 93)	89 (86 to 91)	0.58
ER +	59 (54 to 64)	60 (56 to 64)	0.70
PR +	56 (51 to 61)	59 (54 to 63)	0.12
Lumpectomy	17 (13 to 20)	19 (15 to 22)	0.10
Treatment arm CAF+HDC	57 (51 to 62)	50 (47 to 54)	<0.001
10-year DFS (%)	39 (34 to 44)	43 (38 to 47)	0.02
10-year OS (%)	45 (40 to 51)	48 (44 to 52)	0.09

^1^Pearson's chi-square test

^2^Interquartile range

CAF: cyclophosphamide, doxorubicin fluorouracil; CI: confidence interval; DFS: disease free survival; ER: estrogen receptor; HDC: high dose chemotherapy; LN: lymph nodes; OS: overall survival; PR: progesterone receptor.

Of the 350 subjects in this study, 152 patients were in the standard therapy (CAF) arm and 198 in the high-dose therapy (CAF + HDC) arm. No significant differences in clinical characteristics between the two groups were found (data not shown). The median follow-up for the 136 patients without DFS events by the cutoff date was 9.8 years, with a range of 3.6 to 13.4 years; only 32 (9%) had DFS censored at dates earlier than 8/1/05, and only 12 had DFS censored at dates earlier than 8/1/04. The standard arm had median follow-up of 9.60 years with a recurrence rate (RR) of 63%, while the high-dose therapy arm had a slightly longer median follow-up of 9.9 years with a RR of 48%, though 10-year DFS and OS were not significantly different between the two groups (*P *= 0.08 and *P *= 0.62, respectively). Because there were no significant differences in baseline characteristics or survival between the CAF and CAF+HDC arms in the genotyped cohort, we combined the two groups for survival endpoints and adjusted for treatment arm in multivariable analyses.

Genotypic frequencies by race are shown in Table 2. These are consistent with reported frequencies in the NCI SNP500 database [15]. Because of the paucity of observations for non-white, non-black racial/ethnic groups, we grouped race categories into White, Black, and *Other. *A single missing observation was grouped with the *Other *category. Significant racial differences were seen in CYP2B6, CYP2C9, CYP3A4, CYP3A5, and GSTT1 SNP frequencies; we therefore adjusted for race in the multivariable analysis.

**Table 2:** Genotype frequencies

SNP	Variants	Genotypes	White # (%) (n = 314)	Black # (%) (n = 19)	Other # (%) (n = 17)	*P*-value^1^
CYP2B6	All WT	516 G/G	114 (36)	4 (21)	4 (23)	0.14
(rs2279343,		785 A/A				
rs3211371,		1459 C/C				
rs3745274)	Any Var	Any Var	189 (60)	15 (79)	11 (65)	
	Missing		11 (4)	0 (0)	2 (12)	
CYP2C9	All Wild-	430 C/C	176 (56)	17 (89)	9 (53)	0.06
(rs1799853,	type	1075 A/A				
rs1057910)	Any Var	Any Var	107 (34)	2 (11)	7 (41)	
	Missing		31 (10)	0 (0)	1 (6)	
CYP2D6*4	*1/*1	A/A	19 (6)	0 (0)	0 (0)	0.81
(rs3892097)	*1/*4	G/A	86 (27)	4 (21)	4 (27)	
	*4/*4	G/G	198 (63)	15 (79)	11 (63)	
	Missing		11 (4)	0 (0)		
CYP3A4*1B	*1A/*1A	A/A	281 (89)	5 (26)	13 (76)	<0.001
(rs2740574)	*1A/*1B	G/A	20 (6)	7 (37)	2 (12)	
	*1B/*1B	G/G	3 (1)	7 (37)	0 (0)	
	Missing		10 (3)	0 (0)	2 (12)	
CYP3A5*3	*1/*1	A/A	4 (1)	9 (47)	0 (0)	<0.001
(rs776746)	*1/*3	G/A	39 (12)	8 (42)	5 (29)	
	*3/*3	G/G	260 (83)	1 (5)	11 (65)	
	Missing		11 (4)	1 (5)	1 (6)	
CYP3A5*6	*1/*1	G/G	307 (98)	15 (79)	16 (94)	<0.001
(rs10264272)	*1/*6	A/G	1 (<1)	4 (21)	0 (0)	
	*6/*6	A/A	0 (0)	0 (0)	0 (0)	
	Missing		6 (2)	0 (0)	1 (6)	
GSTM1	Non-null		135 (43)	11 (58)	9 (53)	0.09
	Null		171 (54)	7 (37)	6 (35)	
	Missing		8 (3)	1 (5)	2 (12)	
GSTT1	Non-null		254 (81)	10 (53)	13 (76)	0.02
	Null		51 (16)	8 (42)	2 (12)	
GSTT1	Missing		9 (3)	1 (5)	2 (12)	
GSTP1	All WT	Ex 5-24A/A	146 (47)	6 (32)	7 (41)	0.11
(rs1695, rs1138272)		Ex 6+5 C/C				
	Any Var	Any Variant	160 (51)	13 (68)	8 (47)	
	Missing		8 (3)	0 (0)	2 (1)	
Combined CYP-GST genotype	Favorable		0 (0)	0 (0)	0 (0)	0.06
groups	Intermediate		186 (60)	15 (79)	6 (35)	
	Unfavorable		102 (32)	3 (16)	8 (47)	
	Missing		26 (8)	1 (5)	3 (18)	

^1 ^Exact Pearson's or Chi-square test

SNP: single nucleotide polymorphism

Table 3 shows unadjusted hazard ratios for DFS and OS. CYP3A4*1B variants were associated with decreased DFS compared to *1B wild-type, while GSTM1 null genotypes were associated with improved DFS and OS compared to those with non-null genotype. The prespecified unfavorable CYP/GST genotype group had decreased DFS and OS when compared to the intermediate and favorable groups, though this was not statistically significant.

**Table 3:** Unadjusted models for DFS and OS^1^

Gene/Clinical	Comparison	Unadjusted DFS		Unadjusted OS	
		**HR (95% CI)**	**Wald *P*-value**	**HR (95% CI)**	**Wald *P*-value**
		
**CYP2B6**	**Any variant vs. All WT**	1.10 (0.83, 1.46)	0.51	1.12 (0.83, 1.52)	0.46
**CYP2C9**	**Any variant vs. All WT**	0.97 (0.72, 1.29)	0.82	1.01 (0.75, 1.37)	0.94
**CYP2D6**	**G/A vs. A/A**	1.07 (0.57, 2.03)	0.83	1.30 (0.64, 2.62)	0.46
	**G/G vs. A/A**	0.89 (0.48, 1.65)	0.72	1.01 (0.51, 1.99)	0.98
**CYP3A4**	*1B/*1A vs. *1A/*1A	**1.70 (1.10, 2.63)**	**0.02**	1.21 (0.74, 1.96)	0.45
	*1B/*1B vs *1A/*1A	1.96 (0.96, 3.98)	0.07	1.64 (0.77, 3.50)	0.20
**CYP3A5**	*3/*1 vs. *1/*1	0.76 (0.36, 1.59)	0.47	1.18 (0.49, 2.84)	0.72
	*3/*3 vs. *1/*1	0.74 (0.38, 1.45)	0.38	1.18 (0.52, 2.67)	0.69
**CYP3A5**	*6/*1 vs. *1/*1	2.71 (1.01, 7.32)	0.05	2.47 (0.92, 6.66)	0.07
**GSTM1**	**Null vs. Non-null**	**0.71 (0.54, 0.93)**	**0.02**	**0.71 (0.53, 0.94)**	**0.02**
**GSTT1**	**Null vs. Non-null**	1.41 (1.00, 1.97)	0.05	1.31 (0.91, 1.88)	0.14
**GSTP1**	**Any Var vs. All WT**	1.03 (0.79, 1.36)	0.81	1.18 (0.88, 1.57)	0.27
**CYP-GST genotype groups**	**Unfavorable vs. Intermediate or Favorable**	1.29 (0.97-1.72)	0.08	1.32 (0.98-1.79)	0.07
**Lymph node**	**Continuous**	1.02 (1.00, 1.04)	0.06	1.02 (1.00, 1.04)	0.10
**Tumor size**	**Continuous**	1.00 (1.00, 1.01)	0.36	1.00 (1.00, 1.01)	0.13
**Age**	**Continuous**	0.99 (0.97, 1.00)	0.10	0.99 (0.97, 1.01)	0.28
**ER**	**Pos vs. Neg**	0.79 (0.60, 1.04)	0.10	0.78 (0.58, 1.04)	0.10
**PR**	**Pos vs. Neg**	0.82 (0.62, 1.07)	0.15	0.88 (0.66, 1.17)	0.37
**Race**	**Black vs. White**	1.44 (0.84, 2.48)	0.19	1.41 (0.80, 2.49)	0.23
	**Other vs. White**	1.49 (0.83, 2.68)	0.18	1.20 (0.64, 2.28)	0.57
**Arm**	**CAF+HDC vs. CAF**	0.79 (0.60, 1.03)	0.08	0.93 (0.70, 1.23)	0.59

^1^Cox Regression

CAF: cyclophosphamide, doxorubicin, fluorouracil; DFS: disease free survival; ER: estrogen receptor; HDC: high dose chemotherapy; HR: hazard ratio; PR: progesterone receptor; OS: overall survival

Adjusted hazard ratios (HRs) for DFS and OS were determined for the full multivariable models that included the collapsed dichotomous CYP2B6, CYP2C9, and GSTP1 variables, as well as CYP3A4, CYP3A5 (*3 and *6), GSTM1, and GSTT1. This model also included age, number of positive lymph nodes, tumor size, PR status, race, and treatment arm. Applying the Grambsch-Therneau test for proportional hazards to the fully-adjusted DFS and OS models, estrogen receptor status showed significant evidence of non-proportionality for both outcomes (DFS *P *= 0.003; OS *P *< 0.001). Thus, stratified multivariable Cox models were computed using ER status as a stratification variable to allow for differing underlying hazards in the two estrogen receptor status groups. In the full model for DFS, women heterozygous for the CYP3A4 *1B variant had significantly worse DFS than those who were wild-type (*1A/*1A; hazard ratio [HR] 2.79; 95% CI 1.52, 5.14) (Table 4). Women with the null GSTM1 genotype did not have significantly improved DFS or OS in the full models. Of the clinical variables, the number of lymph nodes (HR 1.02, 95% CI 1.00, 1.04) and the treatment arm (CAF+HDC vs. CAF HR 0.66, 95% CI 0.48, 0.91) remained significantly associated with DFS (Table 4).

**Table 4:** Multivariable cox regression model for DFS

Gene/Clinical	Comparison	Adjusted DFS	
		
		HR (95% CI)	Wald *P*-value
**CYP2B6**	**Any variant vs. All WT**	1.06 (0.77, 1.46)	0.72
**CYP2C9**	**Any variant vs. All WT**	1.11 (0.80, 1.53)	0.53
**CYP2D6**	**G/A vs. A/A**	0.94 (0.46, 1.94)	0.87
	**G/G vs. A/A**	0.99 (0.50, 1.99)	0.99
**CYP3A4**	*1B/*1A vs. *1A/*1A	**2.79 (1.52, 5.14)**	**0.001**
	*1B/*1B vs *1A/*1A	2.67 (0.86, 8.34)	0.09
**CYP3A5**	*3/*1 vs. *1/*1	1.18 (0.47, 2.98)	0.72
	*3/*3 vs. *1/*1	2.09 (0.79, 5.50)	0.14
**CYP3A5**	*6/*1 vs. *1/*1	1.37 (0.39, 4.79)	0.62
**GSTM1**	**Null vs. Non-null**	0.78 (0.57, 1.06)	0.12
**GSTT1**	**Null vs. Non-null**	1.32 (0.86, 2.01)	0.21
**GSTP1**	**Any Var vs. All WT**	0.94 (0.69, 1.28)	0.69
**Lymph node**	**Continuous**	**1.02 (1.00, 1.04)**	**0.03**
**Tumor size**	**Continuous**	1.00 (0.97, 1.01)	0.46
**Age**	**Continuous**	0.99 (0.97, 1.01)	0.46
**ER**	**Pos vs. Neg**	--	--
**PR**	**Pos vs. Neg**	1.01 (0.69, 1.50)	0.95
**Race**	**Black vs. White**	1.08 (0.52, 2.25)	0.84
	**Other vs. White**	1.84 (0.91, 3.70)	0.09
**Arm**	**CAF+HDC vs. CAF**	**0.66 (0.48, 0.91)**	**0.01**

CAF: cyclophosphamide, doxorubicin, fluorouracil; DFS: disease free survival; ER: estrogen receptor; HDC: high dose chemotherapy; HR: hazard ratio; PR: progesterone receptor

**Figure 2:**
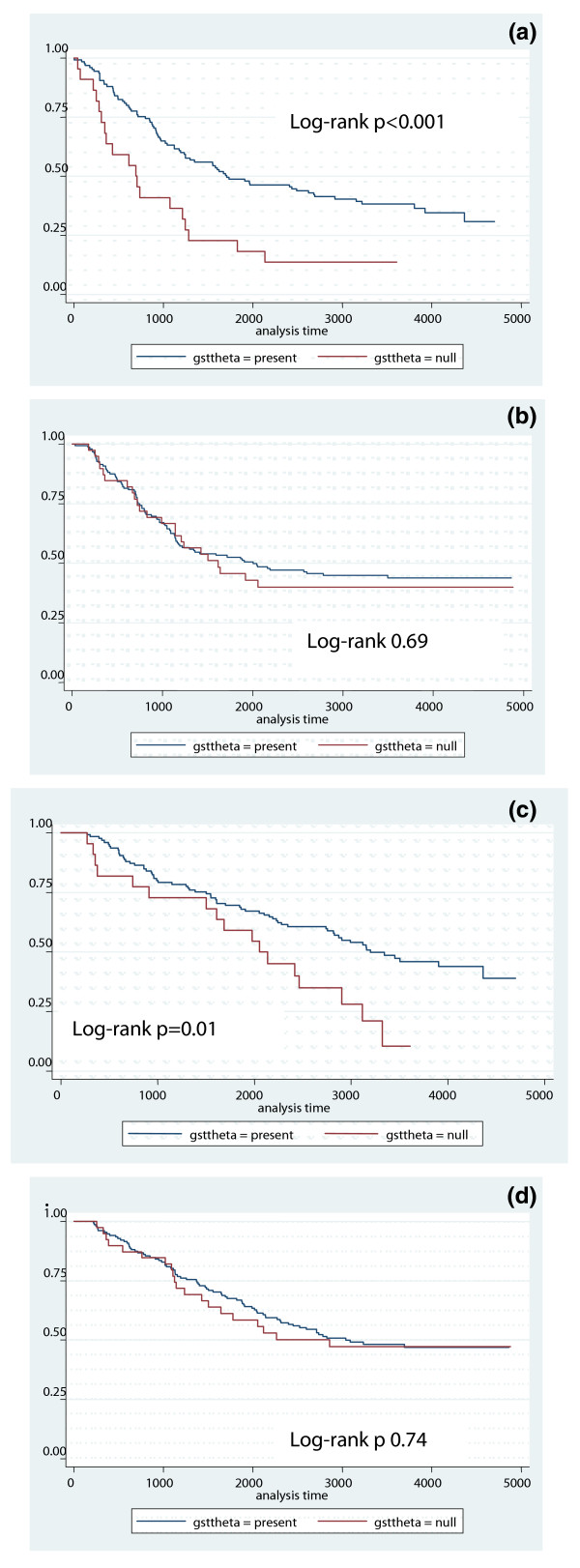
**Stratified Analysis GSTT1 genotype and treatment arm for DFS (2a, b) and OS (2c, d)**. **a) **Arm: CAF; Endpoint: DFS. **b) **Arm: CAF+HDC; Endpoint DFS. **c) **Arm: CAF; Endpoint: OS. **d) **Arm: CAF+HDC; Endpoint: OS.

Because the treatment arm was of significance in the full DFS model, we performed stratified analyses of DFS and OS by treatment arm for each genotype to look for interactions. Treatment arm appeared to modify the relationship between genotype and outcome only for GSTT1 (Figure 2). While a significant difference in DFS by this genotype was seen in the standard therapy (CAF) arm (Adjusted DFS HR 1.95, *P *= 0.053), it was not seen in the high-dose arm (Adjusted DFS HR 0.91, *P *= 0.72). Notably, there have been no relapses or deaths in the high-dose arm beyond the time of median follow-up, whereas this appears not to be the case for the low-dose arm, in which failures have continued to occur with longer follow-up. The interaction term *P*-values for DFS was *P *= 0.04, revealing that for DFS, there was a significant interaction between GSTT1 genotype and dose of cyclophosphamide. This interaction was not seen in the analysis of overall survival

## Discussion

We found that women who received cyclophosphamide-based adjuvant chemotherapy for breast cancer and had the CYP3A4 *1B*/*1A genotype had significantly worse DFS than those who were CYP3A4 *1A/*1A wild-type (HR 2.44, 95% CI 1.52, 5.14). These findings support our hypothesis that reduced phase I enzyme activity (via the CYP3A4*1B polymorphism) leads to a poorer outcome after cyclophosphamide-based adjuvant chemotherapy, presumably as a result of slower activation of cyclophosphamide to HCY. Women with the GST-T1 null genotype had significantly better DFS and OS than those without the variant, though this effect was limited to patients on the standard-dose therapy arm (adjusted DFS HR 1.95, *P *= 0.053)

These data confirm, in a much larger, multicenter patient population in which half of all patients received standard doses of chemotherapy, earlier published data by our group and others examining the effects of cyclophosphamide DME SNPs on breast cancer outcomes. We previously published a single institution study of SNPs in CYP3A4, 3A5*3, 3A5*6, GSTM1 and GSTT1 in which a model utilizing *a*-*priori*-defined genotype combinations showed that patients with an *unfavorable *SNP profile (consisting of either a CYP3A4*1B variant or a CYP3A5*3 variant and wild-type GST T1 and M1) had a significantly increased odds of death compared to those with the favorable genotype (HR 4.6, *P *= 0.045) [6]. While we could not replicate our original analysis using a composite genotype group in this study due to the lack of patients with the *favorable *SNP profile, we did see a non-significant decrement in DFS and OS among those with the *unfavorable *profile compared to those with *intermediate *profiles. A previous study by Petros *et al *also examined a large panel of DME variants, including both phase I and phase II enzymes, as well as drug levels, in 85 metastatic and inflammatory breast cancer patients treated with high-dose cyclophosphamide, cisplatin and carmustine [7], and similarly found that patients with a CYP3A4*1B or CYP3A5*1 variant alleles had higher parent cyclophosphamide levels and significantly worse OS compared to those without the variant (*P *= 0.043), while those with the GSTM1null genotype did significantly better (*P *= 0.041). These data contrast with recent results of a pharmacokinetic study that found no effect of CYP3A4*1B variants on formation of 4-hydroxycyclophosphamide [16]. However, this study of 124 subjects had only three individuals who carried a CYP3A4*1B variant and was thus underpowered to examine this association. Ambrosone and colleagues evaluated the role of GSTM1- and GSTT1-null genotypes on disease-free and overall survival among 251 women who received treatment for incident, primary breast cancer. Adjusting for age, race, and stage at diagnosis, women with null genotypes for GSTM1 and GSTT1 had reduced hazard of death (HR 0.59; 95% CI 0.36 to 0.97; and HR 0.51, 95% CI 0.29 to 0.90, respectively) in relation to those with alleles present. Furthermore, women who were null for both GSTM1 and GSTT1 had one-third the hazard of death of those with alleles for both genes present (adjusted HR, 0.28; 95% CI, 0.11 to 0.70) [17]. Sweeney *et al. *found that women homozygous for genotypes associated with lower activity of GSTP1Val105 or GSTA1*B had better overall survival [18]. A two-SNP haplotype based on CYP3A4*1B and CYP3A5*1A has been associated with docetaxel elimination [19], but as our subjects were not exposed to docetaxel, we were not able to test the association between this haplotype and clinical outcomes.

It is important to note that while this study utilized a cohort of patients enrolled in a multicenter, high-dose therapy trial, our primary study question was not related to high-dose therapy. Our models adjusted for dose to more closely approximate the risk estimates associated with standard-dose therapy. However, we were also able to examine the question of whether higher doses of cyclophosphamide resulted in survival differences by polymorphism. The significantly worse DFS of GST-theta null homozygotes compared to non-null individuals in the CAF arm was not seen in the CAF+HDC arm, in which the two groups have similar DFS. This finding is consistent with our biological hypothesis that higher levels of circulating active drug are associated with improved survival, as suggested by Ambrosone *et al. *[8] and suggests that high-dose chemotherapy was able to surpass some threshold effect for this enzyme, thereby improving disease-free survival for GSTT1 null homozygotes. While it is alternatively possible that homozygous null individuals were less able to detoxify carcinogens and therefore had biologically different breast cancers, as described for other cancers [20,21], the genotypic frequencies in our cohort were not significantly different when compared with the general population, making this explanation unlikely.

Several limitations in this study should be noted. Though only 65% of the patients from INT-0121 had biospecimens available, the genotyped cohort did not differ significantly from the non-genotyped cohort on any clinical variable, with the exception of treatment arm. This imbalance should not impact our results, since effectiveness of treatment arm was not our focus and in the full study cohort, outcome did not differ by treatment arm. We could not assess the effect of population stratification in our cohort because of the paucity of non-Caucasian individuals, limiting sample sizes within strata. However, an analysis restricted to Caucasian individuals did not differ dramatically from the model adjusting for race; therefore, we presented the latter model. In the parsimonious prognostic model, the race variable was not significant. Polymorphisms that could affect metabolism of fluorouracil and doxorubicin might also influence outcomes in this cohort. Thiotepa (a CYP2B6 inducer) did not appear to play a role in the differential effects seen. There was no significant effect of CYP2B6 genotype seen in patients on the standard arm (CAF alone), in the absence of thiotepa, which would have been expected if the thiotepa was ameliorating a true effect of genotype. Furthermore, CYP2D6*4, which was included to account for the possible differential effect of variable tamoxifen metabolism on outcome among patients whose tumors were estrogen-receptor positive, was included in all the models tested, and there was no independent effect of this genotype, nor did it appear to confound the main effects. We chose to limit our candidate gene pool to minimize the risk of false-positive results. In total, we tested 10 SNPs/genotype combinations. We have not adjusted for multiple comparisons, since our relatively large sample size and event rate provide sufficient statistical power for the number of comparisons we made. In genome wide association studies, Bonferroni correction is undertaken to minimize false-positives, but this approach may be an overcorrection in our study where the number of tested exposures is small. We did not include SNPs associated with variable fluorouracil metabolism, as these do not appear to be in linkage disequilibrium with the variants we examined, minimizing unmeasured confounding. Finally, this study would have been strengthened by correlation with serum drug metabolite levels. Unfortunately, samples were not collected prospectively for this purpose, underscoring the need for all trials to include biospecimen collection for pharmacogenetic studies. Similarly, studies such as this one suffer from the lack of information collected on concomitant medications. While several medications may induce CYP activity, potentially modifying the expected effect of genotype, one would anticipate that such effects would be relevant only if a large proportion of the study subjects were taking such medications during chemotherapy.

## Conclusions

In conclusion, this study demonstrates that among women receiving adjuvant chemotherapy for breast cancer, a polymorphism in the cyclophosphamide-metabolizing enzyme CYP3A4 independently contributes to outcomes from cyclophosphamide-based adjuvant breast cancer chemotherapy. Taken together with previous study findings, these results justify prospective studies to further evaluate the relationship between genetic variation, metabolite levels and outcome to determine if tailored pharmacogenetic dosing regimens can improve the efficacy of this therapy.

## Abbreviations

CAF: cyclophosphamide, doxorubicin, fluorouracil; CYP: cytochrome P450; DFS: disease-free survival; DME: drug metabolizing enzymes; ER: estrogen receptor; GST: glutathione S-transferases; HDC: high dose chemotherapy; HR: hazard ratio; OS: overall survival; RR: recurrence rate; SNP: single nucleotide polymorphisms.

## Competing interests

The authors declare that they have no competing interests.

## Authors' contributions

PG participated in study design, data analysis and interpretation, manuscript drafting and revision. IS participated in data analysis and interpretation and manuscript revision. RG participated in data acquisition, data analysis and interpretation, manuscript revision and study supervision. PG participated in study design, data analysis and interpretation and manuscript revision. MH participated in genotyping and data acquisition. RA participated in data acquisition, data interpretation and manuscript revision. WV participated in data acquisition and manuscript revision. MT participated in data acquisition and manuscript revision. TR participated in study design, data interpretation and manuscript revision. AD participated in study design, data analysis and interpretation, manuscript drafting and revision, and study supervision. All authors read and approved the final manuscript.
